# Unveiling the evolutionary pathways of *Ochoterenella*: a new species discovery and its phylogenetic implications

**DOI:** 10.1017/S0031182025100462

**Published:** 2025-06

**Authors:** Gabriel Lima Rebêlo, Jorge Kevin Silva Neves, Fred Gabriel Haick, Ronald Ferreira Jesus, Karina Varella, Luiz Felipe Ferreira Trindade, Leticia de Aguiar da Costa, Fabrícia de Jesus Paiva da Fonseca Sizo, Arnaldo Maldonado Júnior, Carlos Eduardo Costa-Campos, Francisco Tiago Vasconcelos Melo

**Affiliations:** 1 Laboratory of Cellular Biology and Helminthology ‘Profa. Dra. Reinalda Marisa Lanfredi’, Institute of Biological Sciences, Federal University of Pará (UFPA), Belém, PA, Brazil; 2 Laboratory of Biology and Parasitology of Reservoir Wild Mammals, Oswaldo Cruz Institute, Oswaldo Cruz Foundation, Rio de Janeiro, RJ, Brazil; 3 Laboratory of Herpetology, Department of Biological and Health Sciences, Federal University of Amapá (UNIFAP), Macapá, AP, Brazil

**Keywords:** anuran, molecular, nematodes, *Ochoterenella*, Onchocercidae

## Abstract

*Ochoterenella* is a large group of filarial parasites of anurans distributed throughout Central and South America. In the present study, we describe a new species of *Ochoterenella* parasitizing 2 frogs, *Boana geographica* and *Boana multifasciata*, from different localities in the Brazilian Amazon. The main morphological traits that differ *Ochoterenella casiraghii* n. sp. from its congeners are the smaller body size, a shorter cephalic plate, smaller parastomal structures, and the small, short and rounded cuticular bosses on the body of both sexes. The females have a shorter ovejector, and the number of caudal papillae distinguishes males. Pairwise sequence comparisons of the new species reveal a high level of divergence from *Ochoterenella* spp. Our phylogenetic analyses, based on *cox1* and concatenated partial mitochondrial genes, support the monophyly of all subfamilies and genera examined herein. The new species represents the 17th in *the Ochoterenella* genus and a new parasite record for both anuran species. We provide the first ultrastructural description of the species in the genus and establish the phylogenetic relationships of the new species among parasites of amphibians and reptiles from the Onchocercidae.

## Graphical abstract

**Figure:**
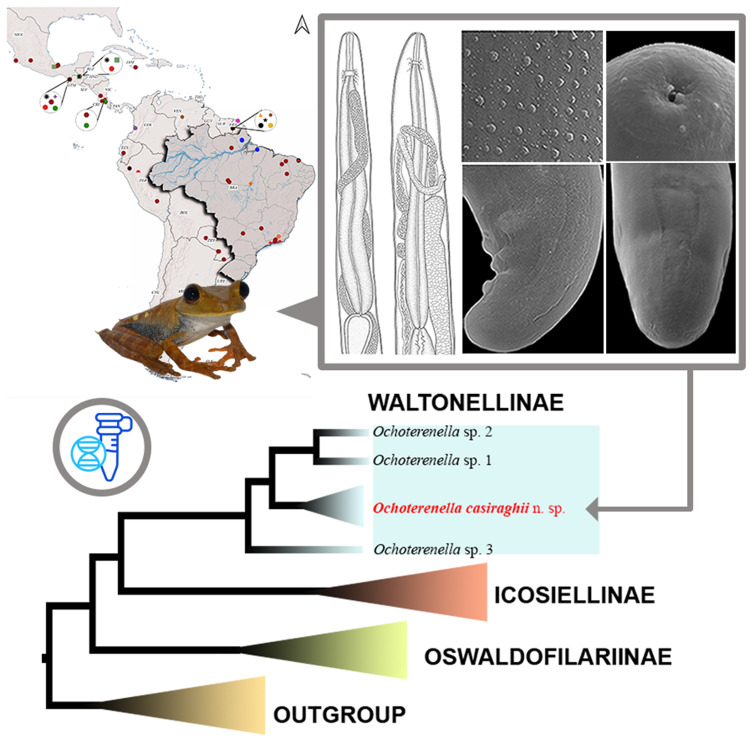

## Introduction

*Ochoterenella* Caballero, 1944 is a large group of filarial parasites of anurans distributed throughout Central and South America (Bain et al., 2013). Currently, 16 species of the genus have been reported parasitizing hosts of the families Bufonidae Gray, 1825, Craugastoridae Hedges, Duellman and Heinicke, 2008, Hylidae Rafinesque, 1815, Leptodactylidae Werner, 1896, Ranidae Rafinesque, 1814 and Strabomantidae Hedges, Duellman and Heinicke, 2008 (Esslinger, 1989; Bursey et al., 2001; Goldberg and Bursey, 2008; Lima et al., 2012; Oliveira et al., 2022).

These nematodes are morphologically similar, which can often lead to confusion and mistakes in species identification (Esslinger, 1986a). Additionally, the males of several *Ochoterenella* spp. remain unknown, and the primary morphological traits used to differentiate species are based on adult females and microfilariae (Lima et al., 2012). Moreover, historically, the authors did not provide details of some species, for example, *Ochoterenella convoluta* Travassos, 1929, *Ochoterenella scalaris* Travassos, 1929 and *Ochoterenella vellardi* Travassos, 1929, in which the descriptions lack even illustrations of the species.

Comprehensive studies using morphological and molecular approaches provided new insights for identification and established phylogenetic relationships between onchocercids and their hosts (Xie et al., 1994; Casiraghi et al., 2001; Bain et al., 2008; Netherlands et al., 2020). However, at the time of those mentioned works, few genetic sequences of *Ochoterenella* were deposited in the genetic database (Casiraghi et al., 2004; Ferri et al., 2011; Lefoulon et al., 2015; Feldman et al., 2020).

Thus, we provide a detailed morphological description of a new filarial worm parasitic in *Boana geographica* (Spix, 1824) and *Boana multifasciata* (Günther, 1859). This species is the first of its genus to be analysed by scanning electron microscopy (SEM). The present work also establishes the phylogenetic relationships of the new species among onchocercid parasites of amphibians and reptiles , based on 2 mitochondrial genes, cytochrome c oxidase 1 (*cox1*) and 12S rDNA.

## Materials and methods

### Host collection and morphological study of parasites

Host specimens were collected from 3 localities in the Amazon biome: 38 specimens of *B. geographica* collected in September 2020 from the ‘Beija-flor Brilho de Fogo’ Extractive Reserve, Pedra Branca do Amapari municipality (0º47′30.6″N, 51°58′42.1″W); 36 specimens of *B. geographica* collected between January and September 2019 from Serra do Navio municipality (0°54′8.68″N, 52°0′19.62″W), both located in Amapá state, Brazil; and 31 specimens of *B. multifasciata* collected between February 2022 and September 2023 from the ‘Centro Nacional de Primatas (CENP)’, Ananindeua municipality (1°22ʹ56.05″S, 48°22ʹ58.13″W), Pará state, Brazil.

The hosts were anaesthetized with sodium thiopental, measured, weighed and necropsied for helminth search (CFMV, 2013). The amphibian hosts are classified according to Frost (2025). Adult filarial nematodes were collected from the body cavity, washed in Petri dishes with saline solution (NaCl 0.9%), killed in heated 70% ethanol and preserved in the same solution at room temperature. For molecular analyses, 3 male specimens were kept in microtubes with 100% ethanol and stored in a freezer at −20 °C.

For morphological and morphometric analyses, the nematodes were hydrated in distilled water, cleared in 50% Amann’s Lactophenol, mounted on temporary slides and examined under an Olympus BX41 microscope (Olympus, Tokyo, Japan) coupled with a drawing tube (without zoom adjustment). The illustrations were prepared using the software CorelDRAW 2021 and processed using Adobe Photoshop Version 21.0.2 software.

We measured morphological characters according to Esslinger (1986a) and Lima et al. (2012). Details of the anterior-end morphology were examined in the apical view, we used 5 specimens of both sexes. For those analyses, we manually sectioned the anterior end with razor blades, mounted the apical end in temporary slides and observed *en face*. Microfilariae samples were extracted from the uterus near the ovijector for further analyses.

The measurements are presented as the values of the holotype followed by the mean and range for the entire type series in parentheses (reported in micrometres unless otherwise indicated) as proposed by Esslinger (1989). The prevalence and mean intensity rates followed Bush et al. (1997) and Reiczigel et al. (2019). The type specimens are deposited in the invertebrate collection of the Museu Paraense Emílio Goeldi (MPEG), Belém, Pará state, Brazil.

Five specimens of both sexes were post-fixed in 1% osmium tetroxide (OsO_4_), dehydrated in an increasing ethanol series and critical-point dried in carbon dioxide (CO_2_). The worms were mounted on metallic stubs, coated with gold-palladium and examined using an SEM Vega3 microscope (TESCAN, Brno, Czech Republic) in the Laboratory of Structural Biology at the Biological Sciences Institute, Federal University of Pará (UFPA), Brazil.

We conducted a bibliographic reference search to compile the records of *Ochoterenella*, using 7 electronic databases (Google, Google Scholar, PubMed, Scielo, Science Direct, Scopus and Web of Science). Species and hosts without specific diagnosis (‘gr.’, ‘af.’ and ‘sp.’) were excluded from our checklist. All records include species, host family, host species, country and locality. Additionally, a map illustrating the distribution of *Ochoterenella* spp. was generated using a spreadsheet and QGIS 3.28 software (Quantum, 2024). This compilation included published records, publicly available data and information from the present study. In the map, we represent through symbols the sex of helminths found in the samples of each species described. The 3 species (*O. convoluta, O. scalaris* and *O. vellardi*) described by Travassos (1929) in Brazil did not have a specified type locality. However, the species are taxonomically valid, and we have considered registers from other localities (Supplementary Table S1).

### Molecular analysis and phylogenetic study

Before conducting molecular analyses, we performed morphological studies using male specimens from each locality. For that, the anterior and posterior portions of the male specimens were cut for light microscopy observations, and the mid-body was used for DNA extraction. The hologenophore (Pleijel et al., 2008) was also preserved and deposited as a voucher in a helminth collection.

Genomic DNA was extracted using the NucleoSpin Tissue kit (Macherey-Nagel, Düren, Germany) according to the manufacturer’s instructions. Polymerase chain reaction (PCR) was conducted to amplify the *cox1* and 12S rDNA, both partial mitochondrial genes, using specific primers and cycle conditions proposed by Casiraghi et al. (2001) and Lefoulon et al. (2015). The resulting amplicons were visualized on a 1.5% agarose gel using GelRed Nucleic Acid Stain (Biotium, Hayward, California, USA) on an ultraviolet light transilluminator.

PCR products were purified using the Illustra GFX PCR DNA and Gel Band kit (GE Healthcare, Chicago, Illinois, USA) according to the manufacturer’s instructions and sequenced using the BigDye Terminator v3.1 Cycle Sequencing kit (Applied Biosystems, USA). Amplicons were sequenced on Applied Biosystems™ 3730 DNA Analyser at the DNA Sequencing Platform of the Oswaldo Cruz Foundation (RPT01A/PDTIS/FIOCRUZ).

For phylogenetic analyses, the forward and reverse sequences obtained were assembled into contigs and edited for ambiguities using the Geneious 7.1.3 software (Kearse et al., 2012). Two datasets were used: the first was based on *the cox1* gene, and the second was a concatenated 12S rDNA and *cox1* sequence. We also prepared a concatenated matrix for both genes in Geneious 7.1.3 software (Kearse et al., 2012). Subsequently, all matrices were aligned and trimmed using Muscle (Edgar, 2004) in Geneious 7.1.3 software (Kearse et al., 2012).

Substitution saturation in the matrices was assessed via the Xia test (Xia et al., 2003; Xia and Lemey, 2009). Both tests were estimated using the DAMBE 5 software package (Xia, 2013). The stop codons were verified according to the translation frame and parameter for invertebrate mitochondrial DNA (translation frame 3, invertebrate mitochondrial table 5) using Geneious 7.1.3 software (Kearse et al., 2012). We excluded from our analyses those sequences that were poorly aligned.

The genetic divergence analysis was conducted using the MEGA11 software package (Kimura, 1980; Tamura et al., 2011). We determined the best-fit evolutionary models in the resulting matrices using the Akaike information criterion in jModelTest software package (Posada, 2008).

Phylogenetic reconstructions were performed using the maximum likelihood (ML) method in RAxML and the Bayesian inference (BI) method in MrBayes (Guindon and Gascuel, 2003; Ronquist and Huelsenbeck, 2003). Both analyses were conducted in the CIPRES Science Gateway (Miller et al., 2010). In the ML analyses, only nodes with a bootstrap percentage (BP) greater than 70% were considered well-supported. In the BI, only nodes with a Bayesian posterior probability (BPP) greater than 90% were considered well-supported.

The trees were visualized and edited in the FigTree v1.3.3 software (Rambaut, 2009). We used *Dipetalonema robini* Petit, Bain and Roussilhon, 1985 (access numbers: KP760183 and KP760329) and *Onchocerca volvulus* Bickel, 1982 (accession numbers: AM749285 and AF015193) as out-groups. The detailed information on onchocercids’ sequences included in the phylogenetic analyses is provided in Table 1.

**Table 1.** Representatives of filarial species and subfamilies, hosts, localities, GenBank accession numbers and references used in phylogenetic analyses

				Accession numbers		
Subfamilies	Species	Definitive host	Locality	COI	12S	Reference
Icosiellinae	*Icosiella* sp.	*Conraua goliath* (Boulenger, 1906)	Cameroon	MH182623	—	Nguiffo et al. (2019)
	*Icosiella neglecta*–h1 (Diesing, 1851)	*Pelophylax ridibundus* (Pallas, 1771)	Ukraine	KP760188	KP760333	Lefoulon et al. (2015)
	*I. neglecta*–(h2, 7, 8)	*P. kurtmuelleri* (Gayda, 1940)	Qazim Pali, Albania	OL351799, OL351804−05	—	Mikulíček et al. (2021)
	*I. neglecta*–h3	*P. kurtmuelleri*	Crkvino, North Macedonia	OL351800	—	Mikulíček et al. (2021)
	*I. neglecta*–h4	*P. kurtmuelleri*	Gjonaj, Kosovo	OL351801	—	Mikulíček et al. (2021)
	*I. neglecta*–(h5, 20)	*P.* kl. *esculentus* (Linnaeus, 1758)	Rusovce, Slovakia	OL351802, OL351816	—	Mikulíček et al. (2021)
	*I. neglecta*–h6	*P.* cf. *bedriagae* (Camerano, 1882)	Garni Canyon, Armenia	OL351803	—	Mikulíček et al. (2021)
	*I. neglecta*–h9	*P. shqipericus* (Hotz, Uzzell, Günther, Tunner, and Heppich, 1987)	Besa, Montenegro	OL351806	—	Mikulíček et al. (2021)
	*I. neglecta*–h10	*P. kurtmuelleri*	Besa, Montenegro	OL351807	—	Mikulíček et al. (2021)
	*I. neglecta*–h11	*P. ridibundus*	Lozenets, Bulgaria	OL351808	—	Mikulíček et al. (2021)
	*I. neglecta*–(h12, 13, 14)	*P.* cf. *bedriagae*	Lebanon, Salima	OL351809−11	—	Mikulíček et al. (2021)
	*I. neglecta*–h15	*P.* cf. *bedriagae*	Dokan, Iraq	OL351812	—	Mikulíček et al. (2021)
	*I. neglecta*–h16	*P.* kl. *esculentus*	France	KP760189	KP760334	Lefoulon et al. (2015)
	*I. neglecta*–h17	*P. ridibundus*	Číčov, Slovakia	OL351813	—	Mikulíček et al. (2021)
	*I. neglecta*–(h18, 21, 22)	*P. ridibundus*	Osli, Hungary	OL351814, OL351817−18	—	Mikulíček et al. (2021)
	*I. neglecta*–h19	*P. lessonae* (Camerano, 1882)	Petržalka, Slovakia	OL351815	—	Mikulíček et al. (2021)
	*I. neglecta*–h23	*P. ridibundus*	Kalinkovo, Slovakia	OL351819	—	Mikulíček et al. (2021)
	*I. neglecta*–h24	*P. ridibundus*	Miklósmajor, Hungary	OL351820	—	Mikulíček et al. (2021)
	*I. neglecta*–h25	*P. kurtmuelleri*	Geoponika, Greece	OL351821	—	Mikulíček et al. (2021)
	*I. neglecta*–(h26, 30,31)	*P.* kl. *esculentus*	Baka, Slovakia	OL351822, OL351826−27	—	Mikulíček et al. (2021)
	*I. neglecta*–h27	*P. ridibundus*	Albrechtičky, Czechia	OL351823	—	Mikulíček et al. (2021)
	*I. neglecta*–h28	*P. ridibundus*	Baka, Slovakia	OL351824	—	Mikulíček et al. (2021)
	*I. neglecta*–h29	*P. ridibundus*	Gbelce, Slovakia	OL351825	—	Mikulíček et al. (2021)
	*I. neglecta*–h32	*P. lessonae*	Rusovce, Slovakia	OL351828	—	Mikulíček et al. (2021)
	*I. neglecta*–h33	*P.* cf. *bedriagae*	Başkavak, Turkey	OL351829	—	Mikulíček et al. (2021)
Oswaldofilariinae	*Oswaldofilaria chabaudi* (Pereira, Souza and Bain, 2010)	*Tropidurus torquatus* (Wied-Neuwied, 1820)	Brazil	KP760204	KP760350	Mikulíček et al. (2021)
	*Oswaldofilaria petersi* (Bain and Sulahian, 1974)	*Crocodilurus amazonicus* Spix, 1825	Peru	KP760205	KP760351	Lefoulon et al. (2015)
Waltonellinae	*Foleyellides calakmulensis* Velázquez-Urrieta Velarde-Aguilar, Oceguera-Figueroa, and León-Règagnon, 2023	*Lithobates brownorum* (Sanders, 1973)	Campeche, Mexico	OR264545−50, 65−70	—	Velázquez-Urrieta et al. (2023)
	*F. calakmulensis*	*L. brownorum*	Quintana Roo, Mexico	OR264559−64	—	Velázquez-Urrieta et al. (2023)
	*F. calakmulensis*	*L. brownorum*	Yucatan, Mexico	OR264551−58, 71	—	Velázquez-Urrieta et al. (2023)
	*Foleyellides mayenae* Romero-Mayén and León-Règagnnon, 2016	*L. psilonota* (= *Rana psilonota*) (Webb, 2001)	Jalisco, Mexico	KC130675−79	—	Prosser et al. (2013), Velázquez-Urrieta et al. (2023)
	*F. mayenae*	*L. pustulosus*	Nayarit, Mexico	KC130681−86	—	Velázquez-Urrieta et al. (2023)
	*Foleyellides striatus* Esslinger, 1986	*L. megapoda* (Taylor, 1942)	Jalisco, Mexico	MZ662824	—	Velázquez-Urrieta et al. (2023)
	*Foleyellides* sp.1	*L. brownorum*	Campeche, Mexico	OR264573−76	—	Velázquez-Urrieta et al. (2023)
	*Neofoleyellides boerewors* Netherlands, Svitin, Smit and Du Preez, 2020	*Sclerophrys gutturalis* (Power, 1927)	Sodwana, KwaZulu-Natal, South Africa	MN663133	—	Netherlands et al. (2020)
	*N. boerewors*	*S. garmani* (Meek, 1897)	Sodwana, KwaZulu-Natal, South Africa	MN663139	—	Netherlands et al. (2020)
	*Neofoleyellides martini* Kuzmin, Netherlands, Du Preez and Svitin, 2020	*Leptopelis natalensis* (Smith, 1849)	Pinetown, KwaZulu-Natal, South Africa	MW774895	—	Kuzmin et al. (2021)
	*Neofoleyellides steyni* Kuzmin, Netherlands, Du Preez and Svitin, 2020	*Amietia delalandii* (Duméril and Bibron, 1841)	Louis Trichardt, Limpopo Province, South Africa	MW598467	—	Kuzmin et al. (2021)
	*Ochoterenella casiraghii* n. sp. Rebêlo, Neves, Trindade and Melo, 2025	*Boana geographica* (Spix, 1824)	Amapá, Brazil	PV745116, PV743300	PV745838	Present study
	*O. casiraghii* n. sp.	*Boana multifasciata* (Günther, 1859)	Pará, Brazil	PV743299	PV747421	Present study
	*Ochoterenella* sp.1	*Rhinella granulosa* (Spix, 1824)	Venezuela	KP760198	KP760343	Lefoulon et al. (2015)
	*Ochoterenella* sp.2	*R. marina*	Venezuela	KP760199	KP760344	Lefoulon et al. (2015)
	*Ochoterenella* sp.3	*Phyllomedusa bicolor* (Boddaert, 1772)	French Guyana	KP760197	KP760342	Lefoulon et al. (2015)
Onchocercinae (Out-group)	*Dipetalonema robini* Petit, Bain and Roussilhon, 1985	*Lagothrix poeppigii* Schinz, 1844	Peru	KP760183	KP760329	Lefoulon et al. (2015)
	*Onchocerca volvulus* (Leuckart, 1893)	*Homo sapiens* Linnaeus, 1758	Italy	AM749285	AF015193	Ferri et al. (2009)

## Results

### Systematics

**Superfamily**: Filarioidea Weinland, 1858

**Family**: Onchocercidae Leiper, 1911

**Subfamily**: Waltonellinae Bain and Prod’Hon, 1974

**Genus**: *Ochoterenella* Caballero, 1944

**Species**: *Ochoterenella casiraghii* n. sp. Rebêlo, Neves, Trindade and Melo, 2025

### Taxonomic summary

**Type host**: *Boana geographica* (Spix, 1824) (Amphibia: Hylidae: Hylinae).

**Additional host**: *Boana multifasciata* (Günther, 1859) (Amphibia: Hylidae: Hylinae).

**Type locality:** ‘Beija-Flor Brilho de Fogo’ Extractive Reserve, Pedra Branca do Amapari municipality, state of Amapá, Brazil (0º47′30.6″N, 51°58′42.1″W).

**Additional locality**: Cancão Municipal Natural Park, Serra do Navio municipality, state of Amapá, Brazil (0°54′8.68″N, 52°0′19.62″W) and ‘Centro Nacional de Primatas (CENP)’, Ananindeua municipality, state of Pará, Brazil (1°22ʹ56.05″S, 48°22ʹ58.13″W).

**Site of infection**: Coelomic/body cavity.

**Infection parameters:** ‘Beija-Flor Brilho de Fogo’ Extractive Reserve prevalence 65.79% (25 infected hosts out of 38 analysed), mean intensity 6.4 (1–31), mean abundance 4.21 (62 males and 98 females); ‘Cancão’ Municipal Natural Park 8.33% (3 infected hosts out of 36 analysed), mean intensity 6.3 (3–10) and mean abundance 0.53 (5 males and 14 females); and ‘Centro Nacional de Primatas’ 9.67% (3 infected hosts out of 31 analysed), mean intensity 5.33 (2–10) and mean abundance 0.51 (5 males and 11 females).

**Type material**: Holotype, male (MPEG.NEM 000408); allotype, female (MPEG.NEM 000410); and paratypes, 9 males (MPEG.NEM 000407), 9 females MPEG.NEM 000409) and hologenophore (MPEG.NEM 000411) were deposited in the Invertebrate Collection of MPEG, Pará, Brazil.

**Additional material**: Cancão Municipal Natural Park vouchers for 3 males (MPEG.NEM 000412), 5 females (MPEG.NEM 000413) and hologenophore (MPEG.NEM 000414) ‘Centro Nacional de Primatas’ vouchers for 4 males (MPEG.NEM 000415), 10 females (MPEG.NEM 000416) and hologenophore (MPEG.NEM 000417) were deposited at the MPEG, Pará, Brazil.

**GenBank Accession number**: *cox1* (PV745116, PV743299 and PV743300), and 12S rDNA (PV745838 and PV747421)

**ZooBank registration**:The Life Science Identifer for *O. casiraghii* n. sp. is urn:lsid:zoobank.org:pub:7EB50CFC-292D-4704-B5FE-489D0AAAD13C

**Etymology**: The specific epithet honours Dr Maurizio Casiraghi for his valuable contributions to the knowledge of filarial nematodes.

**Figure 1.**
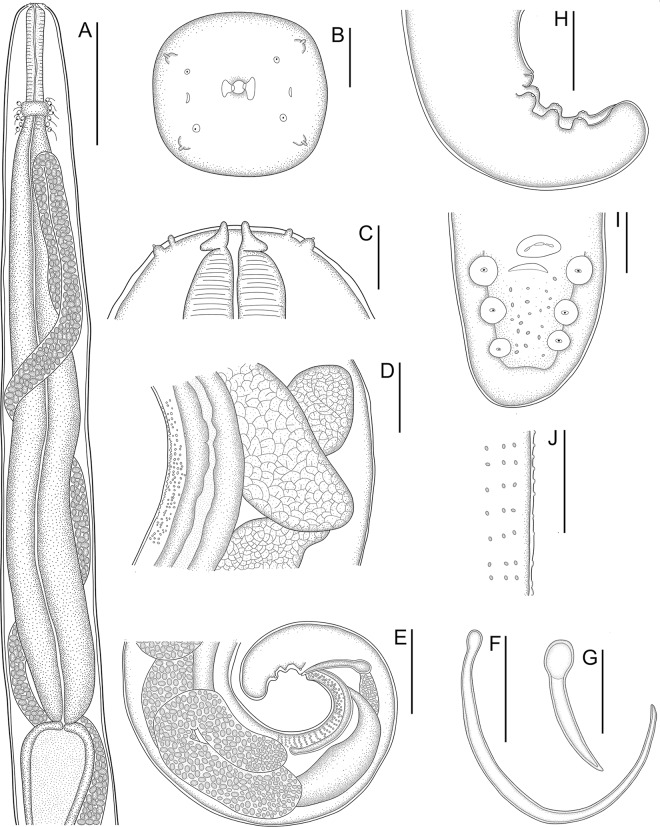
Line drawings of males of *Ochoreterenella casiraghii* n. sp. (A) Anterior end, lateral view; (B) cephalic extremity, apical view; (C) anterior extremity, lateral view; (D) bands of mid-body bosses and testis, lateral view; (E) posterior end, lateral view; (F) left spicule, lateral view; (G) right spicule, ventrolateral view; (H) caudal region, lateral view; (I) caudal region, ventral view; (J) cuticular bosses of the area rugosa, lateral view. Scale bars: A, I = 200 μm; B = 15 μm; C, D = 25 μm; E = 100 μm; F, G, H, J = 50 μm.

**Figure 2.**
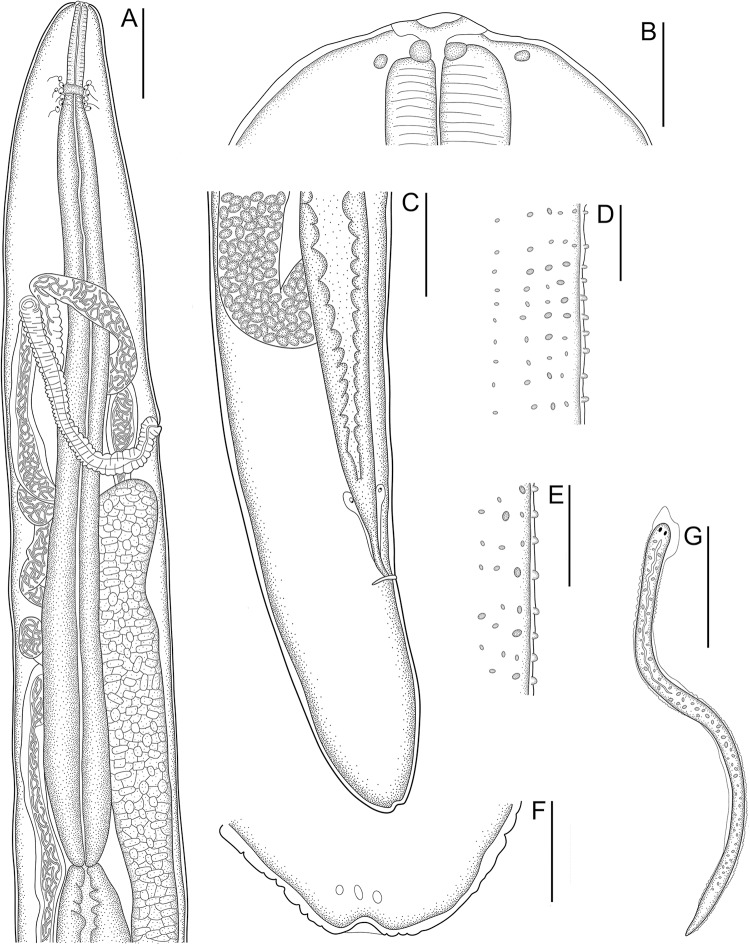
Line drawings of females of *Ochoterenella casiraghii* n. sp. (A) anterior end, lateral view; (B) cephalic extremity, lateral view; (C) posterior end, lateral view; (D) bands of mid-body bosses, lateral view; (E) cuticular bosses on the tail, lateral view; (F) detail of tail tip, lateral view; (G) microfilaria. Scale bars: A = 150 μm; B, C, D, E, G = 25 μm; F = 20 μm.

**Figure 3.**
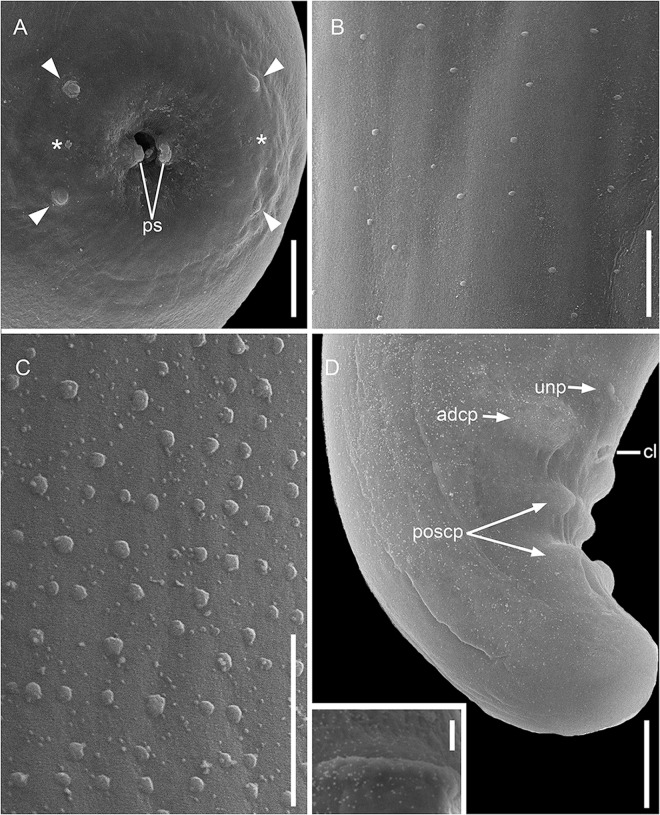
Scanning electron micrographs of males of *Ochoterenella casiraghii* n. sp. (A) Cephalic extremity, apical view (arrowheads: external papillae; asterisk: amphidial pores); (B) detail of mid-body bosses, ventral view; (C) detail of cuticular bosses of the area rugosa, ventral view; (D) caudal region, lateral view. Inset: detail of unpaired papilla. Abbreviations: cl, cloaca; unp, unpaired papilla; adcp; adcloacal papillae; poscp, postcloacal papillae; ps, parastomal structures. Scale bars: A, C = 10 μm; B, D = 20 μm; inset = 2 μm.

**Figure 4.**
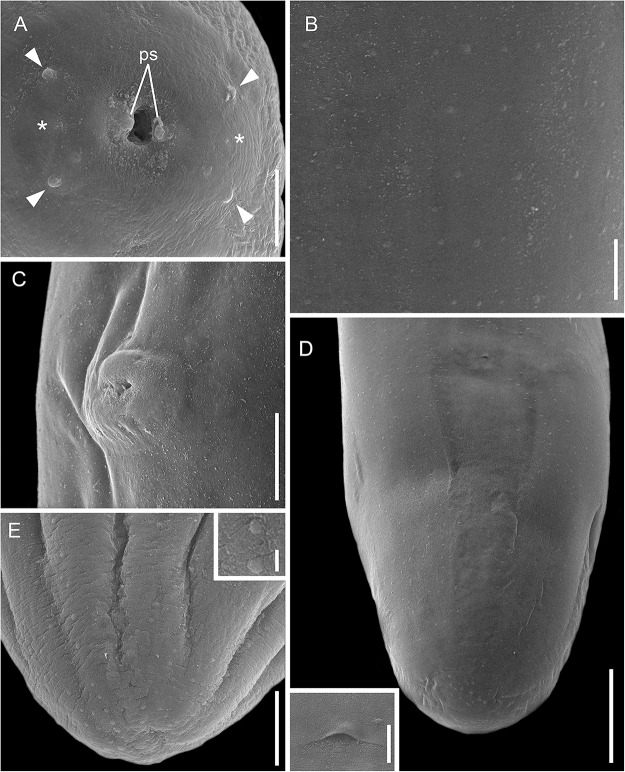
Scanning electron micrographs of females of *Ochoterenella casiraghii* n. sp. (A) Cephalic extremity, apical view (arrowheads: external papillae; asterisk: amphidial pores); (B) detail of mid-body bosses, ventral view; (C) vulva, ventrolateral view; (D) caudal region, ventral view; inset: detail of anus, ventral view; (E) detail of tail tip; inset: detail of bosses of the tail. Abbreviation: ps, parastomal structures. Scale bars: A, B = 10 μm; C, E = 20 μm; D – inset = 2 μm; E – inset: 5 μm.

***General.*** Body filiform, elongated, cylindrical and tapering on both extremities. Widest part posterior to oesophagus–intestinal junction (Figures 1A; 2A). Cuticle thin, caudal and lateral alae absent. Sexual dimorphism evident, females about 2 times longer than males. Cephalic extremity, rounded with flattened end (Figures 1C; 2B). Rectangular cephalic plate with 2 pairs of outer papillae and 2 pairs of internal papillae, each of them with a prominent cuticularized process; a pair of small amphids located laterally (Figures 1B, C; 2B; 3A; 4A). Oral opening circular, surrounded by a pair of small lateral and conspicuous cuticular flap-like parastomal structures (Figures 1B; 3A; 4A). Buccal capsule small and weakly cuticularized, wider than longer (Figures 1C; 2B). Oesophagus filariform divided into short muscular and longer glandular portions (Figures 1A; 2A). Nerve ring encircling muscular oesophagus at level of its posterior quarter (Figures 1A; 2A). Lateral cords present. Cuticular bosses rounded and longitudinally oriented in both sexes (Figures 1D, E, J; 2D, E; 3B, C; 4B, D, E). Microfilariae sheathed (Figure 2G).

***Males*** (based on holotype and 9 paratypes, all adult specimens). Total length 6.7; 7.4 (6.7–8.0) mm. Body width at nerve ring 133; 134 (112–147); width at muscular-glandular oesophagus junction 136; 137 (115–149) and at mid-body 195; 186 (168–211). Cephalic plate 34; 27 (22–34) long × 20; 18 (16–20) wide; length: width ratio 1.4; 1.5 (1.3–1.7). Parastomal structures 2.7 × 1.7. Buccal capsule 8.9; 5.8 (4.2–8.9) in diameter. Outer papillae 3.2; 2.1 (1–3.2) × 2.5; 2.3 (1–4). Oesophagus total length 1.283; 1.361 (1.258–1.464), corresponding to 19.1; 18.5 (16.5–20.2%) of body length. Muscular portion of oesophagus 235; 236 (179–264) × 32; 31 (24–40). Glandular portion of oesophagus 1.048; 1.125 (1.013–1.259) × 120; 116 (96–130). Ratio length of glandular: muscular oesophagus 4.5; 4.8 (3.9–7); ratio width of glandular: muscular oesophagus 3.8; 3.8 (2.8–5.2). Nerve ring located at 219; 215 (176–229) from anterior end; corresponding 3.3; 2.9 (2.7–3.3%) of body length. Testis single, tubular, flexing anteriorly forming loops and bending at glandular part of oesophagus (Figure 1A). Testis runs posteriorly, getting wider and reaching posterior to oesophagus–intestinal junction (Figure 1D). Ejaculatory duct narrower than testis, with a funnel-shaped proximal part (Figure 1E). Small, rounded cuticular bosses present on dorsal and ventral surfaces of the body from oesophagus to caudal region (Figures 1D, J; 3B, C); small bosses initially appear sparse and irregularly arranged, but become more organized and numerous along body, gradually forming evident transverse bands of longitudinally oriented bosses in mid-region of body measuring 1.6; 1.4 (1–1.8) in diameter, distance between bosses 6; 8 (6–13) and distance between bands 10; 11 (8–14). Area rugosa well-developed precloacal, its transverse bands consisted of small, numerous and defined bosses, longitudinally oriented measuring 1.6; 1.8 (1.6–2.6) in diameter, distance between bosses 2.1; 2.5 (1.6–3.2) and distance between bands 2.1; 3.2 (2.1–4.7) (Figures 1J; 3C). Presence of minor bosses and irregularly arranged on caudal region (Figure 1I). Caudal papillae arranged as follows: a single large precloacal plaque-shaped papilla anterior to cloacal aperture; **3** pairs of symmetrically large of sessile papillae: one adcloacal pair and 2 close postcloacal pairs (Figures 1H, I; 3D). Spicules distinctly unequal and dissimilar (Figure 1F, G). Right spicule short and robust, proximal end rounded, expanded and strongly cuticularized at insertion of retractor muscles; distal end sharply pointed and slightly curved ventrally 84; 84 (77–96) long. Left spicule longer and slender, weakly sclerotized, curved ventrally, proximal end rounded, getting gradually tubular and filamentous at distal end 184; 191 (160–242) long; spicular ratio 2.2; 2.3 (2.1–3). Posterior extremity of male, helically coiled with one to 2 turns (Figure 1E). Tail length 84; 94 (74–126); corresponding to 1.3; 1.3 (1–1.7%) of body length. Tail width at cloaca 55; 65 (53–91); length to width ratio 1.5; 1.4 (1.3–1.7).

***Females*** (based on allotype and 9 paratypes, all gravid specimens). Total length 15.5; 13.6 (11.5–15.5) mm. Body width at nerve ring 192; 183 (155–240); at junction of muscular and glandular portions of oesophagus 163; 192 (163–240); at vulva 352; 300 (263–373); and at mid-body 373; 316 (289–373). Cephalic plate 35; 32 (25–36) long × 18; 18 (16–18) wide; ratio of length to width 1.9; 1.8 (1.6–2). Parastomal structures 3.2 × 1.7. Buccal capsule 9; 7.3 (4.7–10) in diameter. Outer papillae 2.6; 2.4 (1.6–3.7) × 3.1; 2.6 (1.6–4.2). Oesophagus total length 2.025; 1.878 (1.485–2.205), corresponding to 13.1; 13.9 (11.3–17.5%) of body length. Muscular portion of oesophagus 221; 261 (221–301) × 39; 39 (32–63). Glandular portion of oesophagus 1.808; 1.617 (1.485–1.952) × 128; 117 (101–132). Ratio length of glandular to muscular 8.2; 6 (5.4–8.2); ratio width of glandular to muscular 3; 3.1 (2.1–3.8). Nerve ring located at 213; 232 (192–280) from anterior end; corresponding to 1.4; 1.7 (1.4–2.2%) of body length. Intestine broad with wide lumen. Rectum thin, short and cuticularized (Figure 2C). Vulva prominent, transverse (Figures 2A; 4C) and located at level of glandular oesophagus at 1.040; 1.067 (888–1.227) from anterior end; corresponding to 6.7; 7.9 (6.7–9.1) of body length and 51; 57 (47–70%) of total oesophagus length. Ovejector muscular 1.101; 945 (581–1.786) long, extending anteriorly and coiled around glandular oesophagus, not reaching muscular oesophagus end (Figure 2A). Uterus containing tightly coiled microfilariae, forming numerous loops and filling the whole body, but not reaching the caudal region (Figure 2C). Cuticular bosses present on dorsal and ventral surfaces along body (Figures 2D, E; 4B, D). Bands of rounded bosses longitudinally oriented in mid-region 1.6; 1.9 (1.6–2.1) in diameter, distance between bosses 9; 11 (8–13) and distance between bands 15; 15 (11–19). On caudal region, bosses irregularly arranged, with different densities 1.6; 1.7 (1.1–2.6) in diameter. Tail rounded, tip with a small depression at posterior end 205; 341 (205–413) long; corresponding to 1.3; 2.5 (1.3–3.3%) of body length. Tail width at anus 168; 226 (168–264); length to width ratio 1.2; 1.5 (1.2–1.9). Anus on a small cuticular elevation (Figures 2C; 4D).

***Microfilariae*** (Figure 2G) (based on 7 specimens, all extracted from the uterus of one gravid specimen). Body cylindrical 106 (95–121) long. Maximum width 4.6 (4.2–5.2). Anterior end wider, rounded, gradually tapering to posterior end with an attenuated tail tip. Sheath present, prominent in both extremities, exceeding length of microfilaria. Cephalic hook small and difficult to distinguish from terminal expansions, imperceptible. Cephalic space short 4.6 (3.7–6.3) long, with 2 large ovoid nuclei. Refractile granules tiny, seen along entire body.

***Variability***: Values of body and oesophagus length varied between samples. The cuticular bosses on the body and pattern of caudal papillae did not vary among the specimens analysed. The measurements of specimens obtained from different localities are given in Table 2.

**Table 2.** Morphometric data of *Ochoterenella casiraghii* n. sp. parasite of tree frogs from different localities

Locality	‘Pedra Branca do Amapari’, Amapá, Brazil**		‘Serra do Navio’, Amapá, Brazil		‘Ananindeua’, Pará, Brazil			
Host	*Boana geographica*		*Boana geographica*		*Boana multifasciata*		Entire sample	
Characters	Males (*n* = 10)	Females (*n* = 10)	Males (*n* = 3)	Females (*n* = 5)	Males (*n* = 4)	Females (*n* = 10)	Males (*n* = 17)	Females (*n* = 25)
Total length (mm)	7.4 (6.7−8.0)	13.6 (11.5−15.5)	7 (6.3−7.6)	12.3 (10.7−15.6)	11.4 (10.4−11.9)	26.3 (22−30)	8.2 ± 1.8	18 ± 6.5
Body width at nerve ring	134 (112−147)	183 (155−240)	140 (120−165)	169 (155−189)	104 (93−115)	171 (147−189)	133 ± 16	176 ± 20
Body width at glandular-muscular oesophagus junction	137 (115−149)	190 (163−240)	145 (123−173)	174 (155−200)	115 (109−120)	182 (171−202)	187 ± 18	184 ± 19
Body width at vulva	—	300 (263−373)	—	320 (232−349)	—	300 (253−333)	—	304 ± 34
Body width at mid-body	186 (168−211)	316 (289−373)	208 (189−234)	346 (291−381)	173 (160−189)	327 (283−410)	191 ± 17	313 ± 47
Cephalic plate length	27 (22−34)	32 (25−36)	19	25	20	28	23 ± 7.7	29.8 ± 5.4
Cephalic plate width	18 (16−20)	18 (16−18)	13	16	15	18	17.8 ± 2.1	17 ± 1.1
Cephalic plate ratio (length/width)	1.5 (1.3−1.7)	1.8 (1.6−2)	1.5	1.6	1.3	1.6	1.5 ± 0.2	1.7 ± 0.21
Parastomal structures height	2.7	3.2	2.7	3.1	2.7	3.2	2.7 ± 0	3.1 ± 0.06
Parastomal structure width	1.7	1.7	1.6	1.7	1.8	2.5	1.7 ± 0.1	2 ± 0.4
Buccal capsule width	5.8 (4.2−8.9)	7.3 (4.7−10)	5.5 (4.7−6.3)	6 (4.7−7.3)	7.4 (5.2−8.9)	9 (8−10)	6.2 ± 1.6	7.4 ± 2.1
Outer papillae height	2.1 (1−3.2)	2.4 (1.6−3.7)	2.6 (1.6−3.7)	2.9 (2.1−3.7)	2.1	3.8 (2.6−5.1)	2.2 ± 0.8	2.7 ± 1
Outer papillae width	2.3 (1−4)	2.6 (1.6−4.2)	2.6 (1.6−3.7)	3.4 (3.1−3.7)	2.1	5.1 (5.1−5.2)	2.4 ± 0.9	3.1 ± 1.2
Oesophagus length	1.361 (1.258−1.464)	1.878 (1.485−2.205)	1.107 (995−1.205)	1.501 (1.344−1.661)	933 (870−1.064)	1.174 (1.013−1.570)	1.215 ± 205	1.521 ± 372
Muscular oesophagus length	236 (179−264)	261 (221−301)	191 (189−195)	203 (179−221)	234 (219−261)	257 (213−285)	227 ± 26	248 ± 35
Mid-length of muscular oesophagus width	31 (24−40)	39 (32−63)	32 (29−35)	35 (27−43)	36 (32−45)	38 (32−48)	32 ± 5.2	38 ± 7.2
Glandular oesophagus length	1.125 (1.013−1.259)	1.617 (1.485−1.952)	916 (760−1.016)	1.299 (1.163−1.440)	916 (760−1.016)	917 (768−1.301)	988 ± 201	1.273 ± 365
Mid-length of glandular oesophagus width	116 (96−130)	117 (101−132)	106 (96−120)	111 (99−139)	62 (56−67)	77 (59−93)	102 ± 25	100 ± 22
Length of glandular to muscular ratio	4.8 (3.9−7)	6 (5.4−8.2)	4.8 (3.9−5.4)	6.5 (5.6−7.6)	3 (2.8−3.2)	3.6 (2.8−4.8)	4.4 ± 1	5.2 ± 1.6
Width of glandular to muscular ratio	3.8 (2.8−5.2)	3.1 (2.1−3.8)	3.3 (3.2−3.4)	3.2 (2.5−3.7)	1.8 (1.2−2)	2.1 (1.6−2.9)	3.2 ± 1	2.7 ± 0.7
Oesophagus to body ratio (%)	18.5 (16.5−20.2)	13.9 (11.3−17.5)	16 (13−18.4)	12.4 (10.6−13.4)	8.3 (7.3−10.2)	4.7 (4−5.7)	15.7 ± 4.6	9.9 ± 4.7
Nerve ring*	215 (176−229)	232 (192−280)	170 (165–173)	179 (152–203)	210 (189–237)	222 (173–277)	206 ± 23	217 ± 35
Nerve ring to body ratio (%)	2.9 (2.7−3.3)	1.7 (1.4−2.2)	2.4 (2.3−2.6)	1.5 (1.2–.1.7)	1.9 (1.6–.2.3)	0.9 (0.6–.1.2)	2.6 ± 0.5	1.3 ± 0.4
Vulva*	—	1.067 (888−1.227)	—	1.084 (827−1.341)	—	897 (667−1.291)	—	1.002 ± 177
Vulva to oesophagus ratio (%)	—	57 (47−70)	—	72 (62−81)	—	77 (56 −96)	—	62 ± 10
Vulva to body ratio (%)	—	7.9 (6.7−9.1)	—	8.8 (7.7−9.7)	—	3.6 (2.3− 4.4)	—	6.3 ± 2.5
Ovijector length	—	945 (581−1.786)	—	1.273 (947−1.672)	—	1.076 (792−1.360)	—	1.071 ± 394
Diameter of cuticular bosses at mid-body	1.4 (1−1.8)	1.9 (1.6−2.1)	2.1 (1.6−2.6)	(2.1−3.9)	1.8 (1.5−2)	1.3 (1.2−1.4)	1.6 ± 0.4	2 ± 0.7
Distance between bosses at mid-region	8 (6−13)	11 (8−13)	9 (7−11)	9 (6−11)	8.1 (6−10)	10 (9−11)	8.2 ± 2.3	10.4 ± 2
Distance between bands at mid-region	11 (8−14)	15 (11−19)	11 (9−12)	16 (13−19)	8.5 (6−10)	11 (10−12)	10.5 ± 2	14.8 ± 2.9
Diameter of cuticular bosses on tail region/area rugosa	1.8 (1.6−2.6)	1.7 (1.1−2.6)	1.8 (1.6−2.1)	2.9 (2.6−3.5)	1.7 (1−2)	1.1 (1.0−1.2)	1.8 ± 0.3	1.8 ± 0.7
Distance between bosses on tail region/area rugosa	2.5 (1.6−3.2)	Irregular	2.3 (1.6−3.1)	Irregular	3.4 (3−4)	Irregular	2.7 ± 0.7	Irregular
Distance between bands of bosses on tail region/area rugosa	3.2 (2.1−4.7)	Irregular	3.1 (2.1−4.2)	Irregular	4.4 (3−5.8)	Irregular	3.5 ± 1.1	Irregular
Caudal papillae arrangement: precloacal/adcloacal	1^a^ /2^b^	—	1^a^ /2^b^	—	1^a^ /2^b^	—	1^a^ /2^b^	—
Caudal papillae arrangement: postcloacal	[2 + 2]	—	[2 + 2]	—	[2 + 2]	—	[2 + 2]	—
Right spicule length	84 (77− 96)	—	80 (77−83)	—	84 (79−87)	—	83 ± 5.4	—
Left spicule length	191 (160−242)	—	175 (151−197)	—	235 (191−292)	—	198 ± 38	—
Spicular ratio (left/right)	2.3 (2.1–3)	—	2.1 (1.8–2.4)	—	2.8 (2.3–3.7)	—	2.4 ± 0.4	—
Tail length	94 (74−126)	341 (205−413)	90 (79−104)	296 (216−344)	92 (84−100)	209 (160−304)	93 ± 13	279 ± 81
Tail width at anus	65 (53−91)	226 (168−264)	72 (65−76)	220 (187−285)	65 (60−73)	170 (140−206)	66 ± 10	202 ± 38
Tail length to width at anus ratio	1.4 (1.3−1.7)	1.5 (1.2−1.9)	1.3 (1.1−1.6)	1.4 (1.1−1.7)	1.4 (1.2−1.7)	1.3 (1−1.5)	1.4 ± 0.2	1.4 ± 0.24
Tail length to body ratio (%)	1.3 (1−1.7)	2.5 (1.3−3.3)	1.3 (1.1−1.4)	2.5 (1.9−3)	0.8 (0.7−9)	0.9 (0.5−1.2)	1.2 ± 0.3	1.8 ± 0.9

All measurements are in micrometres unless otherwise indicated (

a single papillae,

b paired papillae,

* from anterior end and **type series).

### Remarks

The new species was assigned to *Ochoterenella* based on molecular data and the following morphological traits referred by Esslinger (1986a, b) and Lima et al. (2012): oral opening circular surrounded by 2 cuticularized flap-like parastomal structures, distinct buccal capsule, cephalic plate with 4 pairs of articulated papillae, bands of longitudinally oriented bosses in mid-body present in both sexes, absence of lateral and caudal alae; males exhibit unequal and dissimilar spicules; females with vulva located at glandular oesophagus region and sheathed microfilariae.

According to Esslinger (1989), the species of *Ochoterenella* differ in the number, size and position of the cuticular bosses on females. Although males of *Ochoterenella* spp. are often unknown, their morphological characteristics help distinguish species, such as the shape and arrangement of cuticular bosses, the size of spicules and the pattern of caudal papillae.

The females of the new species have short mid-body bosses measuring less than 8 μm. This characteristic resembles *Ochoterenella esslingeri* Souza-Lima and Bain, 2012 (Brazil), described from *Bokermannohyla luctuosa* (Pombal and Haddad, 1993)*; Ochoterenella complicata* Esslinger, 1989 (Colombia); *Ochoterenella dufourae* Bain, Kim and Petiti, 1979 (Guyana); and *Ochoterenella guyanensis* Bain and Prod’Hon, 1974 (Guyana), all of them were described from *Rhinella marina* (Linnaeus, 1758) (Bain and Prod’Hon, 1974; Bain et al., 1979; Esslinger, 1989; Lima et al., 2012). However, *Ochoterenella casiraghii* n. sp. is smaller than *O. dufourae* in body dimensions (11.5–15.5 mm length × 289–373 wide in *O. casiraghii* n. sp. vs 32–44 mm × 560 in *O. dufourae*), cephalic plate (25–36 × 16–18 in *O. casiraghii* n. sp. vs 42 × 30 in *O. dufourae*), individual mid-body bosses (1.6–2.1 in *O. casiraghii* n. sp. vs 4–7 in *O. dufourae*), distance between them (8–13 in *O. casiraghii* n. sp. vs 6–50 in *O. dufourae*) and distance between bands (11–19 in *O. casiraghii* n. sp. vs 30–80 in *O. dufourae*). The tail of the new species has a rounded tip (abruptly attenuated tip, nearly truncate in *O. dufourae*), and microfilariae exhibit a wider anterior end than mid-body (as wide as mid-body in *O. dufourae*), with attenuated posterior end (slightly attenuated in *O. dufourae*).

*Ochoterenella casiraghii* n. sp. can be easily distinguished from *O. esslingeri* by the relative position of the vulva that in the new species is at the oesophagus glandular region, while it is at the intestinal region in *O. esslingeri* (888–1.227 in *O. casiraghii* n. sp. vs 1.672–2.360 in *O. esslingeri*). Additionally, the presence of mid-body bosses is restricted to the posterior region in *O. esslingeri*. The new species is smaller than *O. esslingeri* in body length (11.5–15.5 mm in *O. casiraghii* n. sp. vs 34.5–37.7 mm in *O. esslingeri*), cephalic plate (25–36 × 16–18 in *O. casiraghii* n. sp. vs 53–58 × 30–36 in *O. esslingeri*), body width at vulva (263–373 in *O. casiraghii* n. sp. vs 470–520 in *O. esslingeri*) and ovijector (581–1.786 in *O. casiraghii* n. sp. vs 2.920 in *O. esslingeri*). Furthermore, *Ochoterenella casiraghii* n. sp. has greater values of the oesophagus length to body length ratio (11.5–15.5 mm in *O. casiraghii* n. sp. vs 4.8–6.5 mm in *O. esslingeri*) and vulva to body length ratio (6.7–9.1 in *O. casiraghii* n. sp. vs 3.2–4.3 in *O. esslingeri*).

The new species is smaller compared to *O. guyanensis* in body dimensions (11.5–15.5 mm × 289–373 in *O. casiraghii* n. sp. vs 26.0–45.0 mm × 450 in *O. guyanensis*), cephalic plate (25–36 × 16–18 in *O. casiraghii* n. sp. vs 78 × 50 in *O. guyanensis*), ovejector (581–1.786 in *O. casiraghii* n. sp. vs 2.450 in *O. guyanensis*) and tail length (205–413 in *O. casiraghii* n. sp. vs 640 in *O. guyanensis*). Additionally, in *O. casiraghii* n. sp. mid-body bosses are rounded (rectangular in *O. guyanensis*), individual bosses are smaller (1.6–2.1 in *O. casiraghii* n. sp. vs 5 in *O. guyanensis*), more distant between each other (8–13 in *O. casiraghii* n. sp. vs 4–5 in *O. guyanensis*) and the distance between each band is smaller (11–19 in *O. casiraghii* n. sp. vs 30–35 in *O. guyanensis*). The microfilariae in *O. casiraghii* n. sp. are smaller (95–121 in *O. casiraghii* n. sp. vs 130–190 in *O. guyanensis*), with a wider anterior end than mid-body (slightly attenuated in *O. guyanensis*) and an attenuated posterior end (rounded tip in *O. guyanensis*).

Although *O. casiraghii* n. sp. resembles *O. complicata* in diameter of mid-body bosses, the new species differs in their rounded shape (thin and slightly expanded in *O. complicata*), closest distance between bosses (8–13 in *O. casiraghii* n. sp. vs 18–27 in *O. complicata*) and bands (11–19 in *O. casiraghii* n. sp. vs 26–37 in *O. complicata*). *Ochoterenella casiraghii* n. sp. is smaller than *O. complicata* in body length, (11.5–15.5 mm in *O. casiraghii* n. sp. vs 27–35 mm in *O. complicata*), cephalic plate (25–36 × 16–18 in *O. casiraghii* n. sp. vs 32–50 × 19–26 in *O. complicata*) and parastomal structures (3.2 × 1.7 in *O. casiraghii* n. sp. vs 3.5–4 × 2 in *O. complicata*). Furthermore, the microfilariae of the new species are wider on the anterior end than on the mid-body (as wide as the mid-body in *O. complicata*), with an attenuated posterior end (rounded tip in *O. complicata*).

Until now, the male specimens are known only for the following species of *Ochoterenella: O. convoluta* (Molin, 1858) Esslinger, 1986, *O. digiticauda* Caballero, 1944, *O. esslingeri, O. figueiroai* Esslinger, 1988, *O. guyanensis, O. oumari* Bain, Kim and Petit, 1979*, O. royi* Bain, Kim and Petit, 1979, *O. scalaris* (Travassos, 1929) Esslinger, 1986 and *O. vellardi* (Travassos, 1929) Esslinger, 1986 (Travassos, 1929; Bain et al., 1979; Esslinger, 1986a, 1988b; Lima et al., 2012). However, the new species can be easily distinguished from all of them by the smallest number of postcloacal papillae in males (2 close pairs in *O. casiraghii* n. sp. vs 3 pairs in other species). Furthermore, the new species is smaller in body size (6.7–8.0 mm in *O. casiraghii* n. sp. vs ranging from 14.9 to 36 mm in the other species), cephalic plate (22–34 × 16–20 in *O. casiraghii* n. sp. vs ranging 34–58 × 26–37 in the other species), individual mid-body bosses (1–1.8 in *O. casiraghii* n. sp. vs ranging from 3 to 14 in the other species), the distance between mid-body bands (8–14 in *O. casiraghii* n. sp. vs ranging from 17 to 63 in the other species) and distance between area rugosa bands (2.1–4.7 in *O. casiraghii* n. sp. vs ranging from 14 to 50 in the other species).

Therefore, a combination of unique characteristics distinguishes the new species from its congeners: a smaller body size, a shorter cephalic plate, fewer parastomal structures, small and short individual bosses present on both sexes and the closest distance between bosses and bands. The females have a shorter ovijector, and males differ in the number and arrangement of caudal papillae: a single precloacal plaque-shaped papilla, one adcloacal pair and only 2 close postcloacal pairs.

### *Notes on the distribution of* Ochoterenella *species*

**Figure 5.**
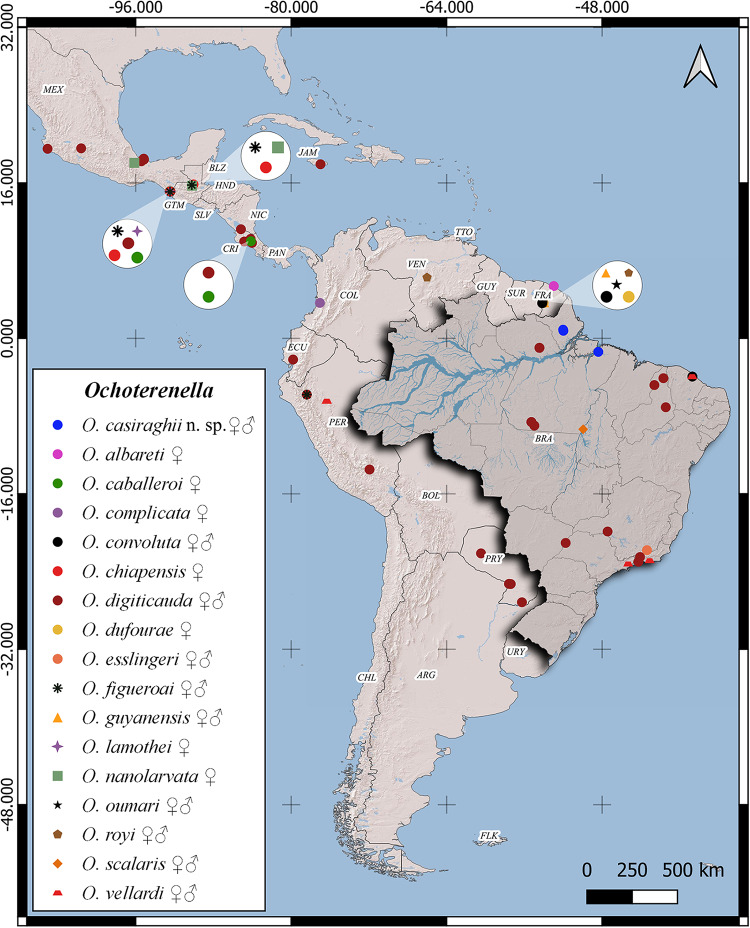
*Ochoterenella* species distribution map. Symbols: ♀ = females; ♂ = males.

Our bibliographic revision revealed that the diversity of *Ochoterenella* comprises 17 taxa parasitizing 31 host species across 11 countries in the Neotropical region. Of those countries, Brazil has the highest number of species (6 taxa), found infecting 20 species of anurans, followed by Mexico (6 taxa and 3 hosts), Guyana (5 taxa and 1 host), Peru (3 taxa and 6 hosts), Guatemala (3 taxa and 1 host), Costa Rica (2 taxa and 4 hosts), Colombia (1 taxon and 1 host), Ecuador (1 taxon and 1 host), Jamaica (1 taxon and 1 host), Paraguay (1 taxon and 1 host) and Venezuela (1 taxon and 1 host) (Figure 5; Supplementary Table S1).

A total of 6 anuran families were recorded: Bufonidae (14 taxa and 5 hosts), Hylidae (5 taxa and 13 hosts), Leptodactylidae (2 taxa and 9 hosts), Craugastoridae (1 taxon and 2 hosts), Ranidae (1 taxon and 2 hosts) and Strabomantidae (1 taxon and 1 host). The giant toad *R. marina* showed the most remarkable species diversity, with 14 taxa recorded. *Ochoterenella digiticauda* is the most common species found, parasitizing 18 hosts from 7 countries. In Brazil, *O. convoluta* and *O. digiticauda* were registered in 5 hosts. We observed that *O. convoluta, O. digiticauda, O. scalaris* and *O. vellardi* were found parasitizing a broad spectrum of host species, while the remaining *Ochoterenella* were recorded in a single host. Males of 7 species are unknown (*O. albareti, O. caballeroi, O. chiapensis, O. complicata, O. dufourae, O. lamothei* and *O. nanolarvata*) (Figure 5; Supplementary Table S1).

### Molecular analyses and phylogenetic study

We obtained 5 sequences, 3 of which were from *cox1* and 2 from 12S, from localities within the Amazon biome (Table 3). The *cox1* matrix resulted in 91 taxa and 325 sites. The model indicated by the JModelTest was HKY + I + G (gamma shape parameter = 0.3070; ln*L* = −2521.7932). The second matrix concatenated included only 11 taxa and 923 sites. The models indicated for the *cox1* and 12S rDNA gene dataset were GTR + I + G (gamma shape parameter *a* = 0.7240; ln*L* = −2071.6143) and TIM3 + G (gamma shape parameter *a* = 0.3410; ln*L* = −1328.9254), respectively. The BI results in both matrices show that the ESSs are robust for all parameters. Xia’s test provided no evidence for substitution saturation in any data matrix.

**Table 3.** Haplotypes obtained from the samples of the present study

				Accession numbers	
Sequences	Definitive host	Locality	State/country	*cox1*	12S
Haplotype 1	*Boana geographica*	‘Beija Flor Brilho de Fogo’ Extractive Reserve, Pedra Braca do Amapari	Amapá, Brazil	PV745116 (660 bp)	PV745838 (465 bp)
Haplotype 2	*Boana multifasciata*	‘Centro Nacional de Primatas’, Ananindeua	Pará, Brazil	PV743299 (650 bp)	PV747421 (474 bp)
Haplotype 3	*Boana geographica*	Cancão Municipal Natural Park, Serra do Navio	Amapá, Brazil	PV743300 (666 bp)	—

**Figure 6.**
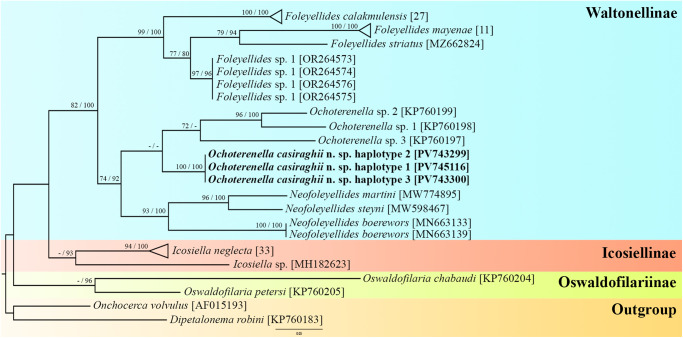
Phylogram of filarid parasites of amphibians and reptiles from the family Onchocercidae based on *cox1* sequences using maximum likelihood (ML) and Bayesian inference (BI). *Dipetalonema robini* and *Onchocerca volvulus* represent the out-groups. GenBank accession numbers follow each taxon. Support values are above or below nodes: posterior probabilities < 90 and bootstrap < 70 are not shown or are represented by a dash. The branch-length scale bar indicates the number of substitutions per site.

**Figure 7.**
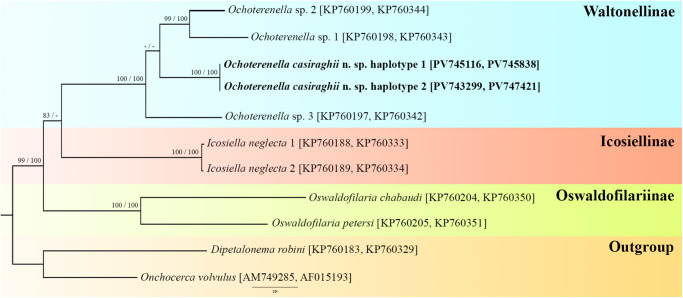
Phylogram of filarid parasites of amphibians and reptiles from the family Onchocercidae based on concatenated datasets of *cox1* and 12S rDNA sequences using maximum likelihood (ML) and Bayesian inference (BI). *Dipetalonema robini* and *Onchocerca volvulus* represent the out-groups. GenBank accession numbers follow each taxon. Support values are above or below nodes: posterior probabilities < 90 and bootstrap < 70 are not shown or are represented by a dash. The branch-length scale bar indicates the number of substitutions per site.

The new sequences are highly divergent from *Ochoterenella* sp.1 (13.14% in *cox1* and 9.06% in 12S rDNA), *Ochoterenella* sp.2 (13.58% in *cox1* and 8.34% in 12S rDNA) and *Ochoterenella* sp.3 (13.61% in *cox1*; 9.72% in 12S rDNA) (Supplementary Tables S2, S3). Both phylogenies revealed 3 main supported clades, corresponding to representatives of the subfamilies Oswaldofilariinae Chabaud and Bain, 1976 (*cox1*: BP = 42, BPP = 96; concatenated: BP = 100, BPP = 100), Icosiellinae Chabaud and Bain, 1976 (*cox1*: BP = 45, BPP = 93; concatenated: BP = 100, BPP = 100) and Waltonellinae (*cox1*: BP = 82, BPP = 100; concatenated: BP = 100, BPP = 100). We also recovered sequences of all genera as monophyletic groups (Figures 6; 7).

Sequences of *Ochoterenella* sp.1 and *Ochoterenella* sp.2 parasite of Bufonidae hosts from Venezuela showed the closest relationships (*cox1*: BP = 96, BPP = 100; concatenated: BP = 99, BPP = 100), while *O. casiraghii* n. sp. parasite of *B. geographica* (Hylidae: Hylinae) from Brazil and *Ochoterenella* sp.3 parasite of *P. bicolor* (Hylidae: Phyllomedusinae) from French Guyana formed separate branches within the clade exclusively formed by *Ochoterenella* species (*cox1*: BP = 63, BPP = 69; concatenated: BP = 100, BPP = 100) (Figures 6; 7).

In the *cox1* phylogenetic tree, the representatives of the Waltonellinae formed 3 major clades: *Foleyellides* Caballero, 1935 (BP = 99, BPP = 99), *Neofoleyellides* Netherlands, Svitin, Smit and Du Preez, 2020 (BP = 93, BPP = 100) and *Ochoterenella* (BP = 63, BPP = 69). *Ochoterenella* is more closely related to *Neofoleyellides* than *Foleyellides* among the other genera. The *Neofoleyellides* clade showed that *N. martini* Netherlands, Svitin, Smit and Du Preez, 2020 and *N. steyni* Netherlands, Svitin, Smit and Du Preez, 2020 (BP = 96, BPP = 100) are positioned closer to each other in the cladogram than *N. boerewors* Netherlands, Svitin, Smit and Du Preez, 2020. Our analyses placed *F. calakmulesis* as a well-supported clade, and a sister group to the clade of *Foleyellides* sp.1 + *F. striatus* (Ochoterena and Caballero, 1932) Caballero, 1935 + *F. mayenae* Romero-Mayén and León-Règagnon, 2016, all parasites of *Lithobates* Fitzinger, 1843 frogs from Mexico (BP = 99, BPP = 100). The subfamily Icosiellinae formed a sister clade to Waltonellinae (BP = 45, BPP = 93), while Oswaldofilariinae (BP = 42, BPP = 96) formed an independent clade of parasites exclusively of reptiles from South America (Figure 6).

The phylogenetic tree reconstructed from concatenated partial mitochondrial sequences recovered similar results to those of the *cox1* phylogeny. Clades of subfamilies and genera remained the same, but Waltonellinae were represented only by *Ochoterenella* (BP = 95, BPP = 99). The Oswaldofilariinae subfamily is the earliest diverging lineage of the in-group analyses. In concatenated trees, most clade support values are higher than those in the *cox1* phylogeny (Figure 7).

## Discussion

The morphological and molecular data strongly support the independent species status of *Ochoterenella* parasitic in 2 hylid frogs from the Brazilian Amazon. Intraspecific variations were observed in body length and the glandular oesophagus (Table 2). Although we did not find genetic divergence among the samples, similar results were also found in *I. neglecta* populations that exhibited morphological variation and high genetic similarity (Kuzmin et al., 2023).

We observed high divergence among the sequences obtained for the new species and its congeners, using the 2 most common molecular markers, *cox1* and 12S rDNA (Supplementary Tables S2, S3). In the case of *cox1*, the values exceeded the threshold of 4.8% used to separate new filarial species (Ferri et al., 2009, 2011; Kuzmin et al., 2023). As previously suggested, both molecular markers are considered suitable for differentiating onchocercid species (Santos et al., 2022).

The *cox1* gene is ideal for resolution at lower taxonomic levels, while the 12S rDNA gene is often concatenated to other mitochondrial genes to maximize the discriminatory power of the nucleotide variability (Casiraghi et al., 2001, 2004; Ferri et al., 2009; Lefoulon et al., 2014; Laidoudi et al., 2021; Mikulíček et al., 2021; Santos et al., 2022). Thus, the genetic divergence observed in our sequences compared to other sequences registered in GenBank reinforces that *O. casiraghii* n. is a new filarid species.

Our phylogenetic analyses of onchocercid parasites of amphibians and reptiles strongly support the monophyly of Oswaldofilariinae, Icosiellinae and Waltonellinae. Previous phylogenetic studies also recovered the clades of these subfamilies, traditionally considered ancient and that diverged before Gondwana’s break-up (Chabaud and Bain, 1994; Bain, 2002; Bain et al., 2008; Lefoulon et al., 2015, 2016; Feldman et al., 2020; Mikulíček et al., 2021; Uni et al., 2022; Velázquez-Urrieta et al., 2023).

In contrast, Kuzmin et al. (2021) and Netherlands et al. (2020), who combined molecular markers, did not recover the monophyly of Waltonellinae as observed in the present study, and Icosiellinae were placed as the sister group to Oswaldofilariinae. These differences observed in the Netherlands et al. (2020) can be explained by the smaller number of genetic regions used; usually, to infer phylogenetic trees at higher taxonomic levels (7 onchocercid subfamilies), this factor can reduce clade resolution. Furthermore, both studies used a different molecular marker (18S rDNA), which changes the number of sequences of taxa in concatenated phylogenetic trees.

Although the 3 subfamilies formed distinct lineages that were closely related, the topologies placed Oswaldofilariinae as the earliest diverging lineage, which evolved independently of the 2 other subfamilies. These findings are supported by previous molecular analyses (Lefoulon et al., 2015, 2016; Feldman et al., 2020; Mikulíček et al., 2021; Uni et al., 2022; Velázquez-Urrieta et al., 2023). Indeed, members of Oswaldofilariinae are parasites of reptiles that have several morphological plesiomorphic traits, including a long oesophagus, large buccal capsule, presence of deirids, the vulva located very far from the anterior end and the infective larvae with longitudinal cuticular body crests (Chabaud and Bain, 1994; Bain et al., 2008; Pereira et al., 2010). Icosiellinae and Waltonellinae form closely related phylogenetic clades, which are restricted to amphibians, and their diversity is primarily a result of the Mesozoic radiations of anuran hosts (Bain and Prod’Hon, 1974; Anderson and Bain, 2009; Bain et al., 2013).

In our study, all genera formed monophyletic groups; these results are similar to most previous morphological and molecular analyses (Esslinger, 1986a, 1986b; Anderson and Bain, 2009; Lefoulon et al., 2015, 2016; Feldman et al., 2020; Netherlands et al., 2020; Kuzmin et al., 2021; Mikulíček et al., 2021; Uni et al., 2022; Wu et al., 2022). Conversely, Velázquez-Urrieta et al. (2023) found that *Foleyellides rhinellae* García-Prieto, Ruiz-Torres, Osorio-Sarabia and Merlo-Serna (2014) grouped within the *Ochoterenella* clade. We did not include this sequence (GenBank access: OR268888 and OR268889) in our comparisons because it was poorly aligned in all matrices. Moreover, our results reinforce the need for a taxonomic reassessment to determine if this species should be transferred to *Ochoterenella*.

*Foleyellides, Neofolleyelides* and *Ochoterenella* comprise genera that are well-supported through both morphological and molecular data; however, their evolutionary relationships remain uncertain. Our results revealed that *Ochoterenella* species appeared to be more closely related to *Neofoleyellides*. In the study by Velázquez-Urrieta et al. (2023), *Ochoterenella* is placed as the sister group of *Foleyellides*. In contrast, Netherlands et al. (2020) and Kuzmin et al. (2021) show *Ochoterenella* and *Foleyellides* as separate branches, with *Neofoleyellides* forming a sister group to the clade composed of Icosiellinae and Oswaldofilariinae. In neither scenario, *Foleyellides* and *Neofoleyellides* were placed into a closely related clade; therefore, molecular analyses suggest that their morphological similarities evolved independently within this group of parasites. As previously suggested by Netherlands et al. (2020), the increased sampling of other species and genera from Waltonellinae will provide a more complete overview of the phylogenetic relationships within this subfamily.

Our results indicate host and geographical associations in *Ochoterenella* species. The affinities amongst *Ochoterenella* species revealed *Ochoterenella* sp.1 (*R. granulosa*) closest to *Ochoterenella* sp.2 (*R. marina*), both parasites of Bufonidae hosts from Venezuela, while *O. casiraghii* n. sp. parasitic in *B. geographica* and *B. multifasciata* from Brazil and *Ochoterenella* sp.3 parasites of *P. bicolor* from French Guiana formed separate branches. Although both species are parasitic in Hylidae hosts, these anurans belong to distinct subfamilies: *B. geographica* and *B. multifasciata* in Hylinae, and *P. bicolor* in Phyllomedusinae. However, only additional sequences of the genus from different localities and anuran taxa will strongly support this hypothesis.

Interestingly, the relationships among *Foleyellides* species parasitizing anurans of the genus *Lithobates* resemble those found by Velázquez-Urrieta et al. (2023). The authors did not show consistent associations in the phylogenetic tree topology related to morphological traits, geographical distribution and host species. In the *Neofoleyellides*, we recovered the same phylogenetic relationships among the 3 species as those reported by Kuzmin et al. (2021), showing *N. martini* and *N. steyni* are closer than *N. boerewors*.

The distribution map showed that *Ochoterenella* species are restricted to Central and South America. In the genus, some species are found on specific hosts, whereas others, such as *O. digiticauda* and *O. vellardi*, have a wide geographic and host distribution, encompassing different hosts and countries. Certain species coexist on the same host and in the same locality, such as Chiapas, Mexico (*O. caballeroi, O. chiapensis, O. digiticauda, O. figueroai* and *O. lamothei*); Guatemala, Guatemala (*O. chiapensis, O. figueroai* and *O. nanolarvata*) and Maripasoula, French Guyana (*O. dufourae, O. guyanensis, O. oumari* and *O. royi*). The fact that some species are known only from a single record in a single host species suggests that their strict host specificity may be overestimated.

The high diversity of *Ochoterenella* in the giant toad, *R. marina*, can be attributed to its widespread geographic range and tolerance of distinct environments, as it inhabits forested areas, semideserts, disturbed habitats and areas surrounding urbanization and roadways. The high number of species with unknown males highlights the importance of new collections for morphological and molecular studies of the genus.

Our study provides the first ultrastructural analyses of the species from the *Ochoterenella* genus. The SEM images displayed a set of essential characteristics used to identify the genus and distinguish species. We observed details of apical structures, the cephalic plate, the external papillae, parastomal structures and amphids. In our analyses, the internal papillae were more challenging to observe, a finding that resembles that of Netherlands et al. (2020) for the *Neofoleyellides*, reinforcing the notion that this structure has poorly developed sensilla at the parasite cuticle. However, it can be easily observed by light microscopy following conspicuous nerves.

The SEM images of the arrangement and shape of cuticular bosses were observed in different regions of both sexes. These notable characteristics are helpful for identification due to their few variations. According to Esslinger (1986a), analyses of other areas of the cuticular bosses on the body should also be considered. Therefore, the electron micrographs obtained helped the examination of these structures, mainly in species with small bosses, as observed herein. Furthermore, the SEM examination confirms the distribution of caudal papillae in males, as well as the details of the vulva and the small cuticular elevation of the anus in females.

Brazil concentrates the highest anuran species richness; however, the diversity of filarial nematodes of anurans appears to be underestimated. Our results strongly support the independent status of *O. casiraghii* n. sp., characterized through light microscopy, SEM and molecular data. The new taxon is the 17th species of *Ochoterenella* and the first species of the genus to be described using ultrastructural analyses. The phylogenetic results indicate that subfamilies and genera form monophyletic clades. The map showed different patterns of distribution; some species may occur concomitantly in specific or broad host ranges. Our results reinforce the importance of detailed morphological and molecular studies in improving our knowledge of the biodiversity, evolutionary history and ecology of this group of anuran parasites.

## Supporting information

Rebêlo et al. supplementary material 1 Rebêlo et al. supplementary material

Rebêlo et al. supplementary material 2 Rebêlo et al. supplementary material

Rebêlo et al. supplementary material 3 Rebêlo et al. supplementary material

## Supplementary material

The supplementary material for this article can be found at https://doi.org/10.1017/S0031182025100462.

## References

[ref1] Anderson RC and Bain O (2009) Spirurida: Diplotriaenoidea, Aproctoidea and Filarioidea. In Anderson RC, Chabaud AG and Willmott S (eds), *Keys to the Nematode Parasites of Vertebrates: Archival Volume*. Wallingford, UK: CABI, pp. 391–448.

[ref2] Bain O (2002) Evolutionary relationships among filarial nematodes. In Rajan K (ed. 5 Springer ), *The Filaria. World Class Parasites*. Boston, MA: Kluwer Academic, pp. 21–28.

[ref3] Bain O, Casiraghi M, Martin C and Uni S (2008) The Nematode Filarioidea: Critical analysis linking molecular and traditional approaches. *Parasite* 15, 342–348. doi: 10.1051/parasite/2008153342 18814705

[ref4] Bain O, Mutafchiev Y and Junker K (2013) Order Spirurida. In Schmidt-Rhaesa A (ed.), *Handbook of Zoology*. Berlin: De Gruyter, 661–732.

[ref5] Bain O and Prod’Hon J (1974) Homogénéité des filaires de batraciens des genres *Waltonella*. *Ochoterenella et Madochotera; Création des Waltonellinae N. Subfam. Annales de Parasitologie Humaine et Comparée* 49, 721–739. doi: 10.1051/parasite/1974496721 4219719

[ref6] Bain O, Prod’Hon J and Petit G (1979) Diversité spécifique des Filaires du genre *Waltonella* coexistant chez *Bufo marinus*. *Bulletin du Muséum National d’Histoire Naturelle* 4, 199–212. doi: 10.5962/p.283225

[ref7] Bursey CR, Goldberg SR and Parmelee JR (2001) Gastrointestinal helminths of 51 species of anurans from Reserva Cuzco Amazónico, Peru. *Comparative Parasitology* 68, 21–35.

[ref8] Bush AO, Lafferty KD, Lotz JM and Shostak AW (1997) Parasitology meets ecology on its own terms: Margolis et al. revisited. *Journal of Parasitology* 83, 575–583. doi: 10.2307/3284227 9267395

[ref9] Casiraghi M, Anderson TJC, Bandi C, Bazzocchi C and Genchi C (2001) A phylogenetic analysis of filarial nematodes: Comparison with the phylogeny of *Wolbachia* endosymbionts. *Parasitology* 122, 93–103. doi: 10.1017/S0031182000007149 11197770

[ref10] Casiraghi M, Bain O, Guerrero R, Martin C, Pocacqua V, Gardner SL, Franceschi A and Bandi C (2004) Mapping the presence of *Wolbachia pipientis* on the phylogeny of filarial nematodes: Evidence for symbiont loss during evolution. *International Journal for Parasitology* 34, 191–203. doi: 10.1016/j.ijpara.2003.10.004 15037105

[ref11] CFMV: Conselho Federal de Medicina Veterinária (2013) *Métodos de Eutanásia. In: Guia Brasileiro de Boas Práticas de Eutanásia em Animais. Comissão de Ética, Bioética e Bem-estar Animal*. Brasília, Distrito Federal: CFMV, pp. 28–29.

[ref12] Chabaud AG and Bain O (1994) The evolutionary expansion of the Spirurida. *International Journal for Parasitology* 24, 1179–1201. doi: 10.1016/0020-7519(94)90190-2 7729976

[ref13] Edgar RC (2004) Muscle: A multiple sequence alignment method with reduced time and space complexity. *BMC Bioinformatics* 5, 1–19. doi: 10.1186/1471-2105-5-113 15318951 PMC517706

[ref14] Esslinger JH (1986a) Redescription of *Ochoterenella digiticauda* Caballero, 1944 (Nematoda: Filarioidea) from the toad, *Bufo marinus*, with a redefinition of the genus *Ochoterenella* caballero, 1944. *Proceedings of the Helminthological Society of Washington* 53, 210–217.

[ref15] Esslinger JH (1986b) Redescription of *Foleyellides striatus* (*Ochoterena* and Caballero, 1932), (Nematoda: Filaroidea) from a Mexican frog, *Rana montezumae*, with reinstatement of the genus *Foleyellides* Caballero, 1935. *Proceedings of the Helminthological Society of Washington* 53, 218–223.

[ref16] Esslinger JH (1988b) *Ochoterenella figueroai* sp. n. and *O. lamothei* sp. n. (Nematoda: Filarioidea) from the Toad *Bufo marinus*. *Proceedings of the Helminthological Society of Washington* 55, 146–154.

[ref17] Esslinger JH (1989) *Ochoterenella complicata* n. sp. (Nematoda: Filarioidea) from the toad *Bufo marinus* in Western Colombia. *Transactions of the American Microscopical Society* 108, 197–203. doi: 10.2307/3226375

[ref18] Feldman SH, Jimenez-Rocha AE, Morales-Acuña JA, León-Bolaños A and Blystone N (2020) Molecular characterization of a new motu *Ochoterenella* (Nematoda: Onchocercidae: Waltonellinae): A case report of a novel subcutaneous filarial parasite infesting a wild-caught red-eyed tree frog (*Agalychnis callidryas*) in Costa Rica 2019. *Integrative Journal of Veterinary Biosciences* 4, 1–7. doi: 10.31038/IJVB.2020423

[ref19] Ferri E, Bain O, Barbuto M, Martin C, Lo N, Uni S, Landmann F, Baccei SG, Guerrero R, Lima SS, Bandi C, Wanji S, Diagne M and Casiraghi M (2011) New insights into the evolution of *Wolbachia* infections in filarial nematodes inferred from a large range of screened species. *PLoS One* 6, e20843. doi: 10.1371/journal.pone.0020843 21731626 PMC3120775

[ref20] Ferri E, Barbuto M, Bain O, Galimberti A, Uni S, Guerrero R, Ferté H, Bandi C, Martin C and Casiraghi M (2009) Integrated taxonomy: Traditional approach and DNA barcoding for the identification of filarioid worms and related parasites (Nematoda). *Frontiers in Zoology* 6, 1–12. doi: 10.1186/1742-9994-6-1 19128479 PMC2657783

[ref21] Frost DR (2025) *Amphibian Species of the World: an Online Reference*. Version 6.1. New York, NY, USA: American Museum of Natural History. https://amphibiansoftheworld.amnh.org (accessed 5 March 2025).

[ref22] Goldberg SR and Bursey CR (2008) Helminths from 10 species of Brachycephalid frogs (Anura: Brachycephalidae) from Costa Rica. *Comparative Parasitology* 75, 255–262. doi: 10.1654/4327.1

[ref23] Guindon S and Gascuel O (2003) A simple, fast, and accurate algorithm to estimate large phylogenies by maximum likelihood. *Systematic Biology* 52, 696–704. doi: 10.1080/10635150390235520 14530136

[ref24] Kearse M, Moir R, Wilson A, Stones-Havas S, Cheung M, Sturrock S and Drummond A (2012) Geneious Basic: An integrated and extendable desktop software platform for the organization and analysis of sequence data. *Bioinformatics* 28, 1647–1649. doi: 10.1093/bioinformatics/bts199 22543367 PMC3371832

[ref25] Kimura M (1980) A simple method for estimating evolutionary rates of base substitutions through comparative studies of nucleotide sequences. *Journal of Molecular Evolution* 16, 111–120. doi: 10.1007/BF01731581 7463489

[ref26] Kuzmin Y, Dmytriieva I and Svitin R (2023) *Icosiella neglecta* (Nematoda, Onchocercidae) in Ukraine: Occurrence, hosts, morphological and molecular characterisation. *Zoodiversity* 57, 75–92. doi: 10.15407/zoo2023.01.075

[ref27] Kuzmin Y, Netherlands EC, du Preez LH and Svitin R (2021) Two new species of *Neofoleyellides* (Nematoda: Onchocercidae) parasitising anuran amphibians in South Africa. *International Journal for Parasitology: Parasites and Wildlife* 14, 298–307. doi: 10.1016/j.ijppaw.2021.02.018 33898231 PMC8056133

[ref28] Laidoudi Y, Lia RP, Mendoza-Roldan JA, Modrý D, De Broucker CA, Mediannikov O, Mediannikov O, Davoust B and Otranto D (2021) *Dipetalonema graciliformis* (Freitas, 1964) from the red-handed tamarins (*Saguinus midas*, Linnaeus, 1758) in French Guiana. *Parasitology* 148, 1353–1359. doi: 10.1017/S0031182021000901 34100346 PMC11010042

[ref29] Lefoulon E, Bain O, Bourret J, Junker K, Guerrero R, Cañizales I, Kuzmin Y, Satoto TBT, Cardenas-Callirgos JM, Lima SS, Raccurt C, Mutafchiev Y, Gavotte L and Martin C (2015) Shaking the tree: Multi-locus sequence typing usurps current Onchocercid (Filarial Nematode) phylogeny. *PLoS Neglected Tropical Diseases* 9, e0004233. doi: 10.1371/journal.pntd.0004233 26588229 PMC4654488

[ref30] Lefoulon E, Bain O, Makepeace BL, d’Haese C, Uni S, Martin C and Gavotte L (2016) Breakdown of coevolution between symbiotic bacteria *Wolbachia* and their filarial hosts. *PeerJ* 4, e1840. doi: 10.7717/peerj.1840 27069790 PMC4824920

[ref31] Lefoulon E, Kuzmin Y, Plantard O, Mutafchiev Y, Otranto D, Martin C and Bain O (2014) Redescription of *Cercopithifilaria rugosicauda* (Böhm & Supperer, 1953) (Spirurida: Onchocercidae) of roe deer, with an emended diagnosis of the genus *Cercopithifilaria* and a genetic characterisation. *Parasitology International* 63, 808–816. doi: 10.1016/j.parint.2014.07.011 25108130

[ref32] Lima SS, Marun B, Alves PV and Bain O (2012) *Ochoterenella esslingeri* n. sp. (Nematoda: Onchocercidae: Waltonellinae) from *Bokermannohyla luctuosa* (Anura: Hylidae) in Minas Gerais, Brazil, with notes on *Paraochoterenella* Purnomo & Bangs, 1999. *Parasite* 19, 341–350. doi: 10.1051/parasite/2012194341 23193518 PMC3671464

[ref33] Mikulíček P, Mešková M, Cyprich M, Jablonski D, Papežík P, Hamidi D, Pekşen ÇA, Vörös J and Benovics M (2021) Weak population‐genetic structure of a widely distributed nematode parasite of frogs in the western Palearctic. *Journal of Zoological Systematics and Evolutionary Research* 59, 1689–1702. doi: 10.1111/jzs.125755

[ref34] Miller MA, Pfeiffer W and Schwartz T (2010) Creating the CIPRES Science Gateway for inference of large phylogenetic trees. In Proceedings of the Gateway Computing Environments Workshop (GCE), November 2010. New Orleans, LA. doi. 10.1109/GCE.2010.5676129

[ref35] Netherlands EC, Svitin R, Cook CA, Smit NJ, Brendonck L, Vanhove MPM and Du Preez LH (2020) *Neofoleyellides boerewors* n. gen. n. sp. (Nematoda: Onchocercidae) parasitising common toads and mosquito vectors: Morphology, life history, experimental transmission and host-vector interaction in situ. *International Journal for Parasitology* 50, 177–194. doi: 10.1016/j.ijpara.2019.11.009 32087248

[ref36] Nguiffo ND, Wondji CS, Pone Wabo J and Mpoame M (2019) Microfilariae infestation of goliath frogs (*Conraua goliath*) from Cameroon. *PLoS One* 14, e0217539. doi: 10.1371/journal.pone.0217539 31141563 PMC6541376

[ref37] Oliveira CR, Mascarenhas W, Batista-Oliveira D, Castro-Araújo K, Ávila RW and Borges-Nojosa DM (2022) Endoparasite community of anurans from an altitudinal rainforest enclave in a Brazilian semiarid area. *Journal of Helminthology* 96, 1–17. doi: 10.1017/S0022149X22000499 35983730

[ref38] Pereira FB, Lima SS and Bain O (2010) *Oswaldofilaria chabaudi* n. sp. (Nematoda: Onchocercidae) from a South American Tropidurid Lizard (Squamata: Iguania) with an update on Oswaldofilariinae. *Parasite* 17, 307–318. doi: 10.1051/parasite/2010174307 21275236

[ref39] Pleijel F, Jondelius U, Norlinder E, Nygren A, Oxelman B, Schander C, Sundberg P and Thollesson M (2008) Phylogenies without roots? A plea for the use of vouchers in molecular phylogenetic studies. *Molecular Phylogenetics & Evolution* 48, 369–371. doi: 10.1016/j.ympev.2008.03.024 18424089

[ref40] Posada D (2008) jModelTest: Phylogenetic model averaging. *Molecular Biology and Evolution* 25, 1253–1256. doi: 10.1093/molbev/msn083 18397919

[ref41] Prosser SW, Velarde-Aguilar MG, León-Règagnon V and Hebert PD (2013) Advancing nematode barcoding: A primer cocktail for the cytochrome c oxidase subunit I gene from vertebrate parasitic nematodes. *Molecular Ecology Resources* 13, 1108–1115. doi: 10.1111/1755-0998.12082 23433320

[ref42] Quantum GIS (2024) QGIS Development Team. QGIS Association. http://www.qgis.org (Accessed 02 february 2025).

[ref43] Rambaut A (2009) Molecular evolution, phylogenetics and epidemiology: Fig-Tree. World Wide Web electronic publication. http://tree.bio.ed.ac.uk/software/figtree/ (Accessed 03 february 2025).

[ref44] Reiczigel J, Marozzi M, Fábián I and Rózsa L (2019) Biostatistic for parasitologists: A primer to quantitative parasitology. *Trends in Parasitology* 35, 277–281. doi: 10.1016/j.pt.2019.01.003 30713051

[ref45] Ronquist F and Huelsenbeck JP (2003) MRBAYES 3: Bayesian phylogenetic inference under mixed models. *Bioinformatics* 19, 1572–1574. doi: 10.1093/bioinformatics/btg180 12912839

[ref46] Santos FAA, Duarte MD, Carvalho CL, Monteiro M, Carvalho P, Mendonça P, Valente PCLG, Sheikhnejad H, Waap H and Gomes J (2022) Genetic and morphological identification of filarial worm from Iberian hare in Portugal. *Scientific Reports* 12, 9310. doi: 10.1038/s41598-022-13354-3 35661130 PMC9166702

[ref47] Tamura K, Peterson D, Peterson N, Stecher G, Nei M and Kumar S (2011) Mega 5: Molecular evolutionary genetics analysis using maximum likelihood, evolutionary distance and maximum parsimony methods. *Molecular Biology and Evolution* 28, 2731–2739. doi: 10.1093/molbev/msr121 21546353 PMC3203626

[ref48] Travassos L (1929) Filaridés des batraciens du Brésil. *Comptes Rendus des Séances de la Societé de Biologie* 100, 967–968.

[ref49] Uni S, Udin ASM, Tan PE, Rodrigues J, Martin C, Junker K, Agatsuma T, Low VL, Lim YA, Saijuntha W, Omar H, Zainuri NA, Fukuda M, Kimura D, Matsubayashi M, Uga S, Takaoka H, Azirun MS and Ramli R (2022) Description and molecular characterisation of *Pelecitus copsychi* Uni, Mat Udin & Martin n. sp. (Nematoda: Onchocercidae) from the white-rumped shama *Copsychus malabaricus* (Scopoli) (Passeriformes: Muscicapidae) of Pahang, Malaysia. *Current Research in Parasitology & Vector-Borne Diseases* 2, 100078. doi: 10.1016/j.crpvbd.2022.100078 36589876 PMC9795348

[ref50] Velázquez-Urrieta Y, Velarde-Aguilar MG, Oceguera-Figueroa A and León-Règagnon V (2023) New species of *Foleyellides* (Nematoda: Onchocercidae: Waltonellinae), parasite of *Lithobates brownorum* (Amphibia: Ranidae) from South-Eastern Mexico and genetic barcodes of the Mexican species of the genus. *Systematic Parasitology* 100, 591–599. doi:10.1007/s11230-023-10108-1 37517005 PMC10613133

[ref51] Wu T, Ma X, Wang F, Xie L, Lv Q, Zeng M, Xu Y, Qin S and Chang Q (2022) First description of the mitogenome features of *Neofoleyellides* genus (Nematoda: Onchocercidae) isolated from a wild bird (*Pyrrhocorax pyrrhocorax*). *Animals* 12, 2854. doi: 10.3390/ani12202854 36290239 PMC9597759

[ref52] Xia X (2013) DAMBE5: A comprehensive software package for data analysis in molecular biology and evolution. *Molecular Biology and Evolution* 30, 1720–1728. doi: 10.1093/molbev/mst064 23564938 PMC3684854

[ref53] Xia X and Lemey P (2009) Assessing substitution saturation with DAMBE. In Lemey P, Salemi M and Vandamme AM (eds), *The Phylogenetic Handbook: A Practical Approach to Phylogenetic Analysis and Hypothesis Testing*. Cambridge: Cambridge University Press, pp. 615–630.

[ref54] Xia X, Xie Z, Salemi M, Chen L and Wang Y (2003) An index of substitution saturation and its application. *Molecular Phylogenetics & Evolution* 26, 1–7. doi: 10.1016/S1055-7903(02)00326-3 12470932

[ref55] Xie H, Bain O and Williams SA (1994) Molecular phylogenetic studies on filarial parasites based on 5S ribosomal spacer sequences. *Parasite* 1, 141–151. doi: 10.1051/parasite/1994012141 9140481

